# Household transmission of respiratory viruses – assessment of viral, individual and household characteristics in a population study of healthy Australian adults

**DOI:** 10.1186/1471-2334-12-345

**Published:** 2012-12-11

**Authors:** James M McCaw, Peter F Howard, Peter C Richmond, Michael Nissen, Theo Sloots, Stephen B Lambert, Michael Lai, Michael Greenberg, Terry Nolan, Jodie McVernon

**Affiliations:** 1 Murdoch Children’s Research Institute & Melbourne School of Population Health, The University of Melbourne, Parkville, Victoria, 3010, Australia; 2 School of Paediatrics and Child Health, University of Western Australia & Telethon Institute for Child Health Research, Princess Margaret Hospital, Perth, WA, Australia; 3 Queensland Paediatric Infectious Diseases Laboratory, Queensland Children’s Medical Research Institute and Sir Albert Sakzewski Virus Research Centre, Queensland Children’s Health Services, University of Queensland, Queensland, Australia; 4 Clinical Research and Development, CSL Limited, Victoria, Australia

**Keywords:** Epidemiology, Transmission, Influenza, human, Influenza vaccines, Respiratory tract infection

## Abstract

**Background:**

Household transmission of influenza-like illness (ILI) may vary with viral and demographic characteristics. We examined the effect of these factors in a population-based sample of adults with ILI.

**Methods:**

We conducted a prospective cohort study in community-dwelling Australian adults nested within an influenza vaccine effectiveness trial. On presentation with ILI, participants were swabbed for a range of respiratory viruses and asked to return a questionnaire collecting details of household members with or without similar symptoms. We used logistic and Poisson regression to assess the key characteristics of household transmission.

**Results:**

258 participants from multi-occupancy households experienced 279 ILI episodes and returned a questionnaire. Of these, 183 were the primary case in the household allowing assessment of factors associated with transmission. Transmission was significantly associated in univariate analyses with female sex (27% vs. 13%, risk ratio (RR) = 2.13 (1.08, 4.21)) and the presence of a child in the house (33% vs. 17%, RR = 1.90 (1.11, 3.26)). The secondary household attack proportion (SHAP) was 0.14, higher if influenza was isolated (RR = 2.1 (1.0, 4.5)). Vaccinated participants who nonetheless became infected with influenza had a higher SHAP (Incidence RR = 5.24 (2.17, 12.6)).

**Conclusions:**

The increased SHAP in households of vaccinated participants who nonetheless had confirmed influenza infection supports the hypothesis that in years of vaccine mismatch, not only is influenza vaccine less protective for the vaccine recipient, but that the population’s immunity is also lower.

## Background

Improved characterisation of the determinants of household transmission of influenza-like illness (ILI) remains an important public health priority, particularly in light of the past decade’s events in which we have witnessed the emergence of severe-acute-respiratory-syndrome (SARS) and the 2009 H1N1 influenza pandemic. The evidence base for pandemic influenza public health interventions such as home-quarantine, provision of antiviral agents for post-exposure prophylaxis, school-closure and vaccination builds upon an appropriate understanding of the patterns and timing of infection within the household unit [1-6].

While influenza viruses, rhinoviruses (HRVs), adenoviruses, respiratory syncytial virus (RSV) and parainfluenza viruses (PIVs) are the most common aetiological agents in acute-respiratory-infection (ARI) episodes [7,8], in 30 – 40% of all ARI episodes no known respiratory virus can be identified [9,10]. This is despite discovery of a number of previously undescribed viruses since 2001 from clinical specimens from the human respiratory tract (human metapneumovirus [11], SARS coronavirus [12], coronavirus NL63 [13], coronavirus HKU1 [14], novel rhinoviruses [8], human bocaviruses [15] and K1 and WU polyomaviruses [16,17]).

Reflecting the need to improve our understanding of household transmission of ARI, the literature examining factors associated with household transmission of influenza [6,18-21] has expanded significantly since the 2009 H1N1 influenza pandemic [22-31], including a systematic review and meta-analysis [32]. Donnelly et al. estimated the serial interval for all ILI (without laboratory confirmation) from case reports during the 2009 pandemic [25]. Only two studies of which we are aware explicitly consider the impact of virus type on infectiousness. Principi et al. found less onwards transmission to household members from influenza-negative than influenza-positive children presenting to a hospital emergency department [33]. Similarly, in a cohort study of ARI in young children, Lambert et. al. observed significant heterogeneity in the proportion of participants’ households in which one or more illness events were observed (ranging from 13% for isolation of hMPV from the child to 61% for isolation of influenza) [34].

Here we report on the household transmission of a range of viruses in a cohort study of healthy, community-dwelling adults reporting symptoms of influenza-like illness (ILI). The study population was sourced from a large, industry sponsored placebo-controlled phase IV efficacy trial of a licensed seasonal trivalent influenza vaccine (Fluvax ®, CSL Ltd), conducted prior to the 2009 pandemic between March and November 2008. In a previous article [35] we have described the viral aetiology of ILI in the cohort, and examined the influence of virus type, host and spatio-temporal factors on disease symptomatology.

## Methods

### Subject recruitment and selection

Full details of subject recruitment and selection for the primary phase IV vaccine efficacy trial have been described previously [35]. Briefly, across 23 study sites in Australia and New Zealand, 7544 healthy adults aged ≥18 to <65 years were recruited for a placebo-controlled trial of a licensed trivalent influenza vaccine (Fluvax®, CSL Ltd) in 2008 (Clinicaltrials.gov #NCT00562484). Study participants were randomized to receive either placebo or vaccine in a 1:2 ratio prior to the southern hemisphere 2008 influenza season. From an available pool of 5624 participants from the primary study at 12 study sites, we consider the 581 persons (adults) who experienced at least one ILI episode – meeting the case definition of at least one respiratory symptom (cough, sore throat, runny nose or nasal congestion) and at least one systemic symptom (fever (oral temperature ≥ 37.8°C), feverishness, chills or myalgia) [35], and who provided written informed consent for participation in the nested cohort-study which required contribution of a valid biological sample (COPAN^TM^ dry flocked swab). Samples were tested for a range of respiratory viruses using a combination of multiplexed and uniplexed conventional and real-time polymerase chain reaction (PCR) assays [35]. Of those, 258 were members of multi-occupancy households, allowing investigation of transmission within the household. Using a non-specific and sensitive ILI definition, they reported an episode of ILI on 279 occasions. For each episode they returned a study questionnaire (Additional file 1) detailing respiratory symptoms (see [35] for details), health seeking behaviour (health care provider consultations, hospital admission, time off work), household characteristics (number of adults (≥18 years) and children (<18 years)) and temporally associated symptoms of ILI (if any) in other household members. From herein, we consider the illness episode as the primary unit of analysis.

The primary phase IV vaccine efficacy trial and nested sub-study were approved by Ethics Committees at all study sites: Royal Children’s Hospital Ethics in Human Research Committee (Victoria), Princess Margaret Hospital for Children, Ethics Committee (Western Australia), Redcliff-Caboolture Health Service District (Queensland), Human Research Ethics Committee Network (Tasmania), Children, Youth and Women’s Health Service Research Ethics Committee (South Australia), Bellberry Ltd (South Australia, Queensland), Cairns District Health Service Cairns Base Hospital Ethics Committee (Queensland).

### Outcome measures and analysis

Virology results were classified into 5 virus groups [35]: influenza (influenza A, influenza B), coronaviruses (OC43, 229E, NL63, HKU1), picornaviruses, other viruses (parainfluenza viruses (1, 2, 3), adenoviruses, human metapneumovirus (hMPV), bocaviruses, RSV and KI and WU polyomaviruses) or none, where *none* indicates that no ‘tested-for’ virus was detected in the participant’s sample, as opposed to a missing sample or inconclusive result. Study participants’ vaccination status (as determined by the primary phase IV trial intervention), physical location (i.e. study site), and socio-demographic characteristics were also recorded.

The relevant outcome measure for this sub-analysis was evidence of transmission within households based on experience of symptomatic illness in at least one other member of the study participant’s household. Study participants were asked to complete the diary on the day following cessation of their own symptoms. They recorded the date of onset of symptomatic ILIs in household members from between 14 days prior to the study participant’s illness through to the day of diary completion. Note that the primary case in the household may or may not be the study participant.

For household ILI events in which the participant was the primary case (183 of 279), transmission may or may not have occurred in the household and so it is statistically valid to develop univariate and multivariate explanatory models. For participants who reported recurrent ILI episodes during the study in which the same virus was isolated, we exclude all but the first episode. Events with co-introduction, defined as onset of symptoms in the participant and one or more household members on the same day, were also excluded. Following these exclusions, 177 episodes remained for analysis.

For household ILI events in which the participant was not the primary case (95 of 279), while we do know who the introducer was for these events (via the questionnaire data), other household ILI events initiated by that introducer that did not involve the participant are unobserved. That is, any events in which a child (or for that matter, any other adult member of the household) introduced an infection that *did not* infect the study participant are not captured by the study protocol. This observation necessitates the exclusion of all household ILI events in which the participant was not the primary case from the analyses.

In the one remaining household ILI event, the status of the participant (primary or not) was unknown, so the episode was excluded from the analysis.

For the 177 episodes in which the participant was the primary case, logistic regression models were used to explore associations between host, demographic or virus variables with any observation of within-household transmission (outcome variable = transmission in household for each recorded ILI episode in a study participant). The secondary household attack proportion (SHAP) was calculated as the proportion of potentially exposed household members (assumed susceptible) experiencing illness, averaged over all recorded episodes. We present descriptive statistics for the SHAP and its variation by virus, participant and demographic variables. Poisson regression models were used to assess the influence of virus, participant and demographic variables on the number of secondary cases within a given household, offset against the number of potentially exposed household members (outcome variable = number of secondary cases in household for each recorded ILI episode in a study participant).

Vaccination status of participants was not included in the primary logistic and Poisson statistical analyses due to its known mitigating effect on the likelihood of influenza acquisition [35]. Investigation of the influence of prior immunisation on influenza transmission in ‘breakthrough cases’ was explored in a secondary analysis by inclusion of a statistical interaction term between vaccination and influenza-identification status.

We make an empirical calculation of the mean time between the onset of symptoms in the primary case and the onset of symptoms in the household contacts (the *serial interval*), for all household ILI events, events in which the participant was the primary case, and events in which influenza was isolated from the participant’s virological sample.

All statistical analyses were conducted in Stata/IC 11.1.

## Results

**Figure 1:**
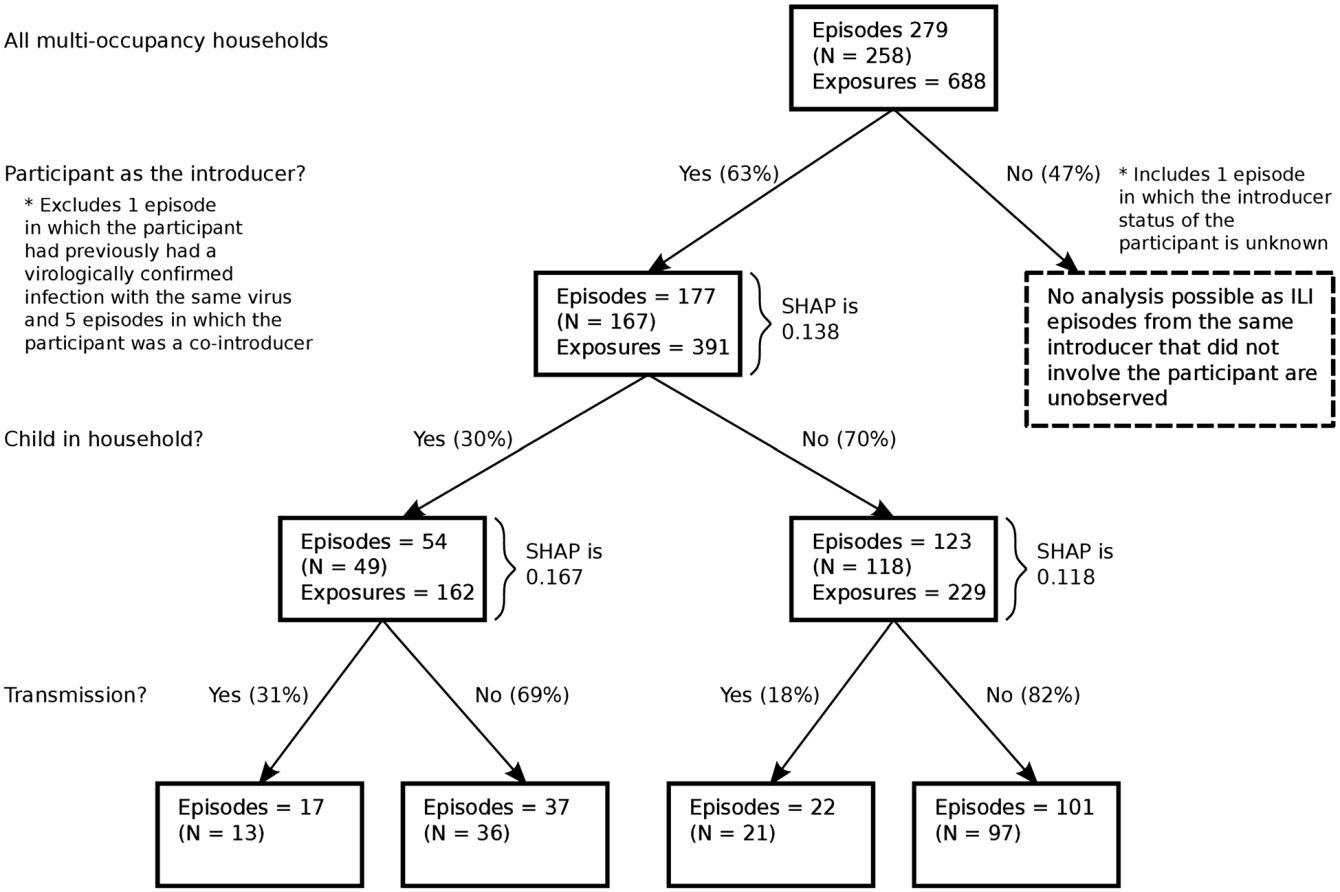
Breakdown of households, by introducing-status of participant, demographic characteristics and evidence of within-household transmission.

Figure 1 reports characteristics of the 258 multi-occupancy households in which transmission did and did not occur. 28 study participants reported two or more ILI episodes during the course of the study. For two participants, who both experienced two episodes, picornavirus was isolated on both occasions. We only retain the first episode for each participant.

**Figure 2:**
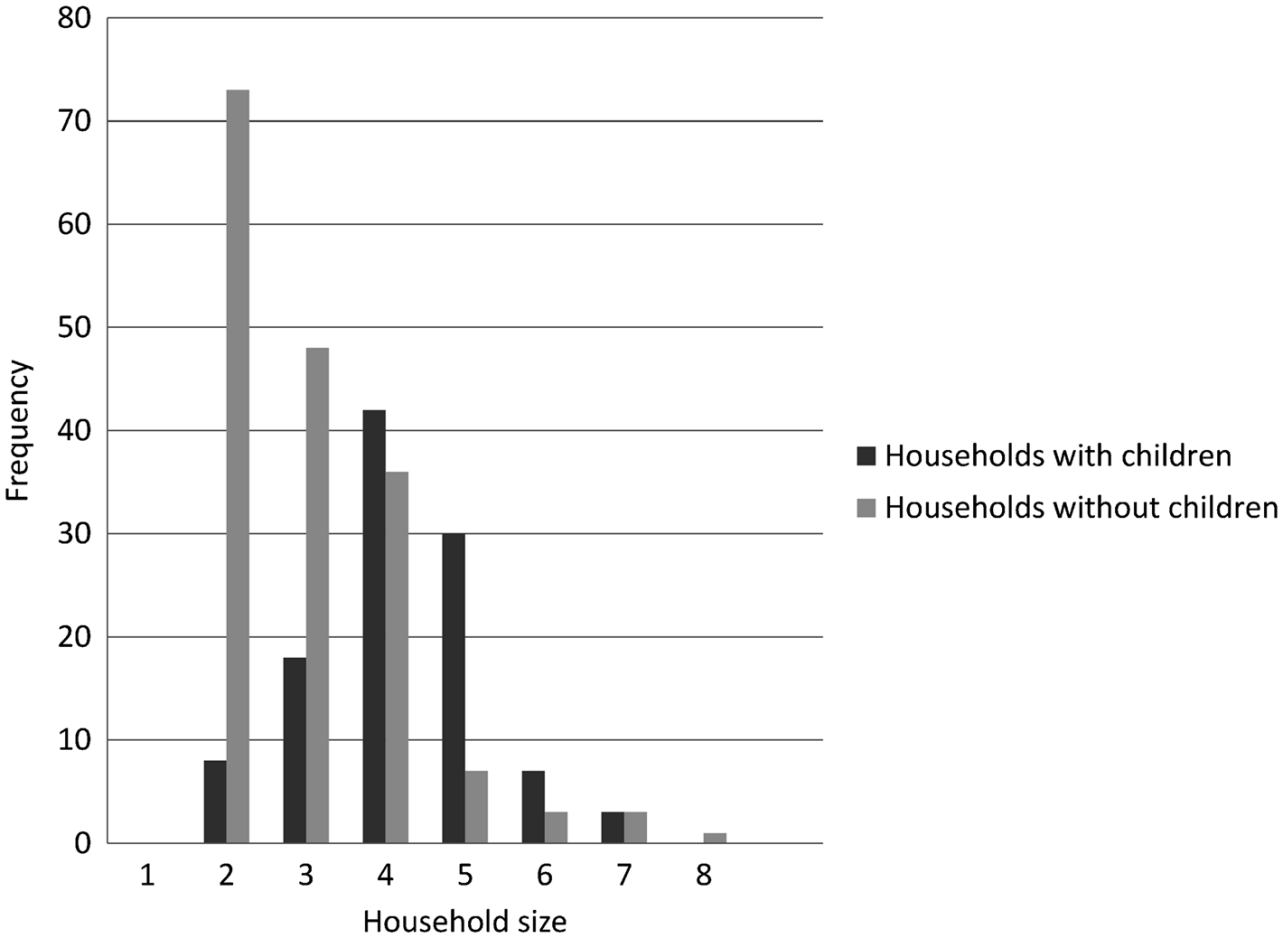
Household size distribution by presence of children (279 episodes).

The distribution of household size is dramatically different based on the absence or presence of children within the household (Figure 2). In households without children, the distribution is left-skewed (mean household size = 3.02, standard deviation (SD) = 1.18, skewness = 1.45), while in households with children there is minimal skew (mean household size = 4.18, SD = 1.11, skewness = 0.103).

### Factors associated with any transmission within the household

The study participant was the primary case in 183 of the 279 recorded household ILI events. One re-introduction of picornavirus into the household and five co-introductions were recorded, leaving 177 episodes for analysis. Transmission occurred in 39 (22.0%) episodes. Stratifying by presence of child (Figure 1), transmission occurred in 17 of 54 (31.5%) households in which at least one child was present and 22 of 123 (17.9%) households without children, a risk-ratio (RR) of 1.76 (1.02, 3.04), *p* = 0.045.

Female sex of the participant was associated with increased transmission: 30 of 108 (27.8%) episodes in women compared to 9 of 69 (13.0%) episodes in men, a risk ratio of 2.13 (1.08, 4.21), *p* = 0.021. The association with female sex remained (at borderline significance) when restricted to households *without* children (123 episodes): 23.3% transmission compared to 10.0% for males, a risk ratio of 2.33 (0.919, 5.90), *p* = 0.059. The risk ratio for female sex in households *with* children (54 episodes) was 1.76 (0.668, 4.66), *p* = 0.224.

Table 1 summarizes the descriptive statistics (and logistic model results) associated with presence or absence of transmission in the household for the 177 household ILI events in which the participant was the primary case.

**Table 1:** Predictors of transmission (participant primay case, 177 household ILI events)

**Explanatory variable**		**Transmission % for group**	**Univariate logistic regression**		**Multivariate logistic regression**	
			**OR (95% CI)**	***p***	**OR (95% CI)**	***P***
Virus Group (cf none detected)	None	20 of 95 (21.1%)	1.00 (def)	-	1.00 (def)	-
	Picornavirus	10 of 40 (25.0%)	1.25 (0.524, 2.98)	0.615	0.996 (0.398, 2.49)	0.994
	Influenza	5 of 17 (29.4%)	1.56 (0.493, 4.95)	0.448	1.87 (0.552, 6.36)	0.314
	Coronavirus	3 of 6 (50.0%)	3.75 (0.703, 20.0)	0.122	2.43 (0.409, 14.4)	0.330
	Other	0 of 8 (0.00%)	-	-	-	-
Sex (cf male)	Male	9 of 69 (13.0%)	1.00 (def)	-	1.00 (def)	-
	Female	30 of 108 (27.8%)	2.56 (1.13, 5.81)	0.024	2.45 (1.01, 5.93)	0.047
Smoking (cf not current smoker)	Not current smoker	36 of 161 (22.4%)	1.00 (def)	-	1.00 (def)	-
	Current smoker	3 of 12 (25.0%)	1.16 (0.298, 4.50)	0.833	1.40 (0.319, 6.12)	0.658
Age (cf 18–24 years)	18-24 years	9 of 47 (19.2%)	1.00 (def)	-	Not included	-
	25-34 years	5 of 28 (17.9%)	0.918 (0.274, 3.08)	0.890	Not included	-
	35-44 years	15 of 37 (40.5%)	2.88 (1.08, 7.66)	0.034	Not included	-
	45-54 years	6 of 36 (16.7%)	0.844 (0.270, 2.64)	0.771	Not included	-
	55-64 years	4 of 29 (13.8%)	0.676 (0.188, 2.43)	0.549	Not included	-
Household size (cf size 2)	2	14 of 64 (21.9%)	1.00 (def)	-	Not included	-
	3	4 of 44 (9.09%)	0.357 (0.109, 1.17)	0.089	Not included	-
	4	11 of 46 (23.9%)	1.22 (0.456, 2.76)	0.801	Not included	-
	5	8 of 17 (47.1%)	3.17 (1.03, 9.75)	0.044	Not included	-
	6	2 of 3 (66.7%)	7.14 (0.602, 84.7)	0.119	Not included	-
	7	0 of 3 (0.00%)	-	-	Not included	-
Presence of child (cf no children in house)	No child in house	22 of 123 (17.9%)	1.00 (def)	-	1.00 (def)	-
	At least one child in house	17 of 54 (31.5%)	2.11 (1.01, 4.41)	0.047	2.63 (1.18, 5.88)	0.018

There is marked co-linearity between the variables ‘presence of child in household’, ‘age-category’ and ‘household size’. For example, respondents aged 35 – 44 years had significantly greater odds of having a child in the household than those aged 18 – 24 years (OR 53.2 (13.0, 217)), while no participant aged more than 55 years lived with a child. The relationship between the household size distribution and presence or absence of children is depicted in Figure 2. We retained ‘presence of child in household’ in the final multivariate model for transmission due to its strong predictive role, intuitive appeal, presumed causal role in our observed (univariate) association with age-category, and previous research indicating an association between transmission and children [18,22,23,29,36]. In the multivariate model, the observed increased risk of transmission with female sex remains (OR = 2.45 (1.01, 5.93), *p* = 0.047). Presence of children in the household is both the strongest and most statistically significant factor associated with transmission (OR = 2.63 (1.18, 5.88), *p* = 0.018).

### Factors associated with the number of secondary household infections

Within 258 multi-occupancy households, 177 *primary-participant* introductions gave rise to 54 secondary cases among 391 potentially exposed individuals, a secondary household attack rate (SHAP) of 0.138. Of 102 exposed children, 22 developed ILI (SHAP = 0.216) compared to 32 of 289 adults (SHAP = 0.110), a risk-ratio of 1.95 (1.19, 3.19), *p* = 0.012. The adult SHAP did not differ by presence of children in the household (0.117 vs. 0.109, RR = 1.07 (0.486, 2.35)).

In households in which the participant was female, 41 secondary infections were reported among 238 exposed household members (SHAP = 0.172), compared with 13 secondary cases among 153 contacts in households in which the participant was male (SHAP = 0.085), a risk-ratio of 2.03 (1.12, 3.66), *p* = 0.016 (2-sided Fisher’s exact). In households with children, 27 secondary infections were reported among 162 exposed household members (SHAP = 0.167), compared with 27 secondary cases among 229 contacts in households without children (SHAP = 0.118), a risk-ratio of 1.41 (0.863, 2.32), *p* = 0.182.

A multivariate Poisson regression model (Table 2) was used to consider the influence of virus group and demographic characteristics on the number of reported secondary cases within a given household, offset against the number of potentially exposed household members. In correspondence with the logistic regression model for transmission, we include presence of children in the household rather than age-category or household size in the model. Consistent with the findings from the multivariate model for transmission and the univariate SHAP analyses, we observe a significant effect of sex. Presence of children in the household, while suggestive of an increase in the SHAP, is not statistically significant. In contrast to the analysis on transmission, we observe a positive association for the number of secondary cases with isolation of influenza (Incidence Risk Ratio (IRR) = 2.11 (0.992, 4.49), *p* = 0.052). Note that if, as an alternative to “no virus detected”, we use picornavirus as the reference group, we again find a positive association with isolation of influenza (IRR = 2.25 (0.960, 5.29, *p* = 0.062) in a multivariate model, full results not shown).

**Table 2:** Predictors of the number of secondary cases in the household, offset against the number of potentially exposed household members (participant primay case, 177 household ILI events)

**Explanatory variable**		**SHAP for group (# episodes)**	**Multivariate Poisson regression**	
			**IRR (95% CI)**	***p***
Virus Group (cf none detected)	None	0.129 (95)	1.00 (def)	-
	Picornavirus	0.143 (40)	0.937 (0.475, 1.85)	0.851
	Influenza	0.238 (17)	2.11 (0.992, 4.49)	0.052
	Coronavirus	0.357 (6)	1.80 (0.670, 4.85)	0.243
	Other	0.000 (8)	-	-
Sex (cf male)	Male	0.085 (69)	1.00 (def)	-
	Female	0.172 (108)	2.12 (1.10, 4.09)	0.025
Smoking (cf not current smoker)	Not current smoker	0.136 (161)	1.00 (def)	-
	Current smoker	0.250 (12)	1.91 (0.738, 4.94)	0.183
Age (cf 18–24 years)	18-24 years	0.106 (47)	Not included	-
	25-34 years	0.115 (28)	Not included	-
	35-44 years	0.235 (37)	Not included	-
	45-54 years	0.095 (36)	Not included	-
	55-64 years	0.100 (29)	Not included	-
Household size (cf size 2)	2	0.219 (64)	Not included	-
	3	0.068 (44)	Not included	-
	4	0.123 (46)	Not included	-
	5	0.206 (17)	Not included	-
	6	0.200 (3)	Not included	-
	7	0.000 (3)	Not included	-
Presence of child (cf no children in house)	No children in house	0.118 (123)	1.00 (def)	-
	At least one child in house	0.167 (54)	1.51 (0.864, 2.65)	0.147

In a secondary analysis, we considered the influence of prior vaccination on the reported number of secondary household cases among participants testing positive for influenza compared with all other participants. In a Poisson model for secondary attacks including a statistical interaction between influenza detection (true/false) and vaccination status (placebo/vaccine), the IRR for influenza positive cases in those receiving placebo was 1.69 (0.421, 6.80), *p* = 0.459. The factor increase (interaction term) for the IRR for vaccinated participants was 3.10 (0.608, 15.8), *p* = 0.174, yielding a net IRR for vaccinated influenza-positive participants relative to vaccinated influenza-negative participants of 5.24 (2.17, 12.6), *p* < 0.001).

### Serial interval

Under the simplifying assumption that the introducer of infection into the household is responsible for all subsequent infections, we may calculate an empiric serial interval, the time from symptom onset in one individual until symptom onset in another. We first consider infections to be related if symptoms are reported within 14 days following onset in the primary case. Across all virus-type isolations, we calculate a mean serial interval of 6.0 days (SD = 3.6) for all household ILI events (where the study participant was the primary case or otherwise), and 5.1 days (SD = 3.2) for the events in which the participant was the primary case. For the five events in which the primary participant had virologically confirmed influenza and transmission occurred, the mean serial interval was 4.5 days (SD = 1.6). If we limited the maximum serial interval to seven days, the mean was reduced to 4.0 days (SD = 1.7) for all household ILI events and 3.9 days (SD = 1.9) for events in which the participant was the primary case. No gaps of greater than seven days occurred for the five events in which the primary participant had virologically confirmed influenza and transmission occurred.

### Sensitivity analysis

In our main analysis, we made two assumptions that we now subject to a sensitivity analysis. Of the 183 events in which our participant was the primary case, 6 were classified as co-introductions as (at least) one other household member recorded symptoms beginning on the same day. As the latent period for respiratory infections may vary from individual to individual, here we exclude a further 4 episodes in which there was a 1 day interval from onset of symptoms in the study participant to onset of symptoms in another household member. The resulting multivariate models (equivalent to Tables 1 and 2) are materially unchanged, with the expected slight reduction in statistical power (data not shown).

A second assumption made was that, for participants who reported multiple ILI episodes during the study period, we only excluded the latter ILI episode where the same respiratory pathogen was isolated on both occasions. However, if we conservatively exclude all ILI episodes except for the first (10 episodes excluded (by virus type: 6 “none”, 1 “picornavirus”, 2 “influenza”, 1 “coronavirus”)), again we find no material change in either the logistic or Poisson analyses (data not shown).

## Discussion

This study, notable in its consideration of a broad range of respiratory pathogens in addition to influenza, demonstrates that household transmission of ILI is most strongly associated with host and demographic factors: female sex and the presence of children within the household (Tables 1 and 2).

The observation that female sex may be associated with increased transmission *in the absence* of children (RR = 2.33 (0.919, 5.90), *p* = 0.059) is novel, perhaps suggesting that females are fundamentally more *infectious*, and not simply more connected to children (in terms of both their susceptibility compared with males if a child introduces infection, and their infectiousness to children if they are the primary household case). Behavioural differences whilst ill may drive such an observation. Alternatively, mechanisms by which influenza pathogenesis is sex dependent have been investigated [37]; whether or not differences extend to infectiousness and susceptibility is not clear. Barbara et al. have recently identified that the reporting of respiratory symptoms may be linked with risk perception [38] and hence gender [39]. Clearly, we cannot exclude the possibility of gender difference in the reporting of within household transmission.

The association between transmission and the presence of children within the household is consistent with many other studies [18,22,23,29,36]. The logistic and Poisson model findings (Tables 1 and 2) are consistent with an increased susceptibility for children. This is further supported by the observed increased SHAP in children compared to adults (0.216 compared to 0.110, a risk-ratio of 1.95 (1.19, 3.19). The SHAP in adults did not differ by whether or not their household contained children, suggesting that other ‘indirect’ effects of children are less likely. As our study design limited the analysis to household events with an adult introducer, we were unable to assess the hypothesis that children may be more infectious than adults.

Our Poisson regression analysis on the number of secondary cases given that the participant was the primary case (Table 2) indicates that isolation of influenza in the introducer of infection to the household is associated with an increase in the number of secondary cases. We explored this finding more deeply using a statistical interaction model. While somewhat limited by sample size, we found that in placebo recipients identification of influenza was not significantly associated with an increase in the number of secondary cases (IRR = 1.69 (0.421, 6.80), *p* = 0.459), while in vaccine recipients the IRR (relative to identification of any other virus, including ‘none’) was 5.24 (2.17, 12.6), *p* < 0.001. Note that our previous analysis confirms that vaccination is associated with a reduced probability of influenza virus identification [35]. Additionally, ‘breakthrough’ influenza cases have similar symptoms compared to unvaccinated individuals [35]. We therefore suggest that our finding of increased transmission may be explained by infection with an influenza virus mismatched to the vaccine-strain (known to be in circulation during the year of study [40]), which furthermore may be relatively antigenically novel and to which household members may be expected to have heightened susceptibility. With no virological samples available from other household members and the small number of vaccinated participants who were infected with influenza we are unable to explore this hypothesis further.

Across all virus types isolated and all household ILI events, and assuming that all secondary cases within the household are directly infected by the introducer, we calculate a serial interval of 6.0 days. Restricting to events in which the participant was the primary case and in which influenza was isolated, we calculate a serial interval of 4.5 days. This simple approach, as taken by others [22,29], cannot account for two important factors: community importation and infection of household members by other non-introducing members (i.e. tertiary cases). While others have partially accounted for these effects [25,29,41], a mechanistically-motivated statistical model is required to fully account for such possibilities, for example as introduced by Cauchemez et al. [23] who determined a serial interval for influenza of 2.6 days (SD = 1.3) compared to 2.9 days if calculated directly from empirical observations. With just 5 events, application of these more advanced model-based techniques is not justified for our data.

Of primary interest for this sub-analysis focussed on *transmission* is the complication introduced by the monitoring and assessment of ILI in an *individual* rather than a household. Ideally, a protocol such as that suggested by Klick et al. would have been employed [42]. The lack of virological assessment of household secondary cases and the broad nature of the question used to establish the secondary case count in each house also contributes to uncertainty with regards to our assignment of temporally associated ILI to within-household transmission. Both of these limitations were an unavoidable consequence of the nesting of the data-collection protocol within a randomized placebo-controlled trial. Furthermore, due to the requested timing for completion of the questionnaire, we cannot exclude the possibility that late onset of secondary (or tertiary etc.) cases may have been missed, particularly if a participant’s experience of symptoms was of short duration. Similarly, because the study protocol and analyses effectively assume that individuals are infectious until the end of their symptoms, any systematic differences (by virus type) in this relationship may influence the results. However the prompt to return the diary upon symptom cessation was in an effort to ensure timely reporting of questionnaire information to minimise recall bias. Conversely, our Poisson model implicitly assumes independence among household members, attributing all household infections to the primary case. More advanced model based methods that account for tertiary (and subsequent) cases and community introduction would be warranted with more complete data sources.

As with all protocols based purely on symptomatic presentation (as opposed to active surveillance for nonclinical signs of infection such as virological or immunological measures [24,42]), we are unable to account for potential sub-clinical infection routes, with potential impact for our assessment of whether or not transmission did occur, the primary case status of our participants and determination of the size of the susceptible pool within a given household. Conversely, taking a non-simulation approach to analysis, we are unable to discount our estimate for the SHAP due to the effects of community introduction into the household, or account for community introduction and tertiary cases in our estimate for the serial interval [23].

Our study sample had an over-representation of females (166 of 258 (64.3%) individuals for the 279 captured episodes; 105 of 167 (62.9%) individuals for the 177 primary-participant introductions). Furthermore, it should be noted that the study population were originally volunteers in a randomized controlled trial and as such more likely to represent a group who were more concerned with their health than the general population. Eligibility was restricted to healthy adults without recognized risk factors for severe influenza infection.

## Conclusions

In the context of a literature focussed on the transmission characteristics of laboratory confirmed influenza, our study is the only one that we know of to systematically explore the relationship between transmission and virus aetiology. The analyses suggest that influenza is more transmissible than other causative agents of ILI, at least when introduced to the household by an adult. Host and demographic factors are also of importance. Further studies combining active surveillance of all household members with specimen collection and testing for a range of respiratory pathogens are warranted to elucidate these relationships.

## Competing interests

PCR has previously served on a scientific advisory board regarding influenza vaccines for CSL Ltd and has received a grant for an investigator initiated epidemiological study of otitis media from GlaxoSmithKline Australia. He has also received travel support for himself and staff employed by the Vaccine Trial Group to attend and present data at scientific meetings from Baxter, GlaxoSmithKline, Sanofi and Pfizer.

MDN has received travel grants from Wyeth Australia to present independent research at international meetings, and currently and previously has been the principal investigator for clinical trials sponsored by Abbott, Baxter, CSL, GSK, MedImmune, Merck, Novartis, Sanofi-Pasteur, Wyeth, and Pfizer. ML is an employee of CSL Limited and has an equity interest in the company.

## Authors’ contributions

JMC, PH and JMV conducted the statistical analyses, provided the primary interpretation of the results and wrote the manuscript. PR was principal investigator on the vaccine efficacy trial within which the sub-study was conducted. TN, JMV, TS, MN, SL and PR conceived the sub-study and secured funding for its conduct, in partnership with CSL Limited represented by ML and MG. JMV coordinated conduct of the study at multiple sites and oversaw collation of the questionnaire data. TS, MN and SL oversaw conduct of and reporting of the virological testing at the Queensland Paediatric Infectious Diseases Laboratory. ML was medical monitor for the main vaccine study and a partner investigator on the sub-study, as was MG. All authors contributed to critical revision of the manuscript and have seen and approved the final version of the manuscript.

## Pre-publication history

The pre-publication history for this paper can be accessed here:

http://www.biomedcentral.com/1471-2334/12/345/prepub

## Supplementary Material

Additional file 1 Illness Visit Questionnaire. Click here for file
