# Supplementary material for: Household transmission of respiratory viruses – assessment of viral, individual and household characteristics in a population study of healthy Australian adults
Source: BMC Infect Dis. 2012 Dec 11;12:345. doi: 10.1186/1471-2334-12-345 (PMC3538067; doi:10.1186/1471-2334-12-345)
Supplement: Additional file 1 — Illness Visit Questionnaire. [file 1471-2334-12-345-S1.pdf]

Participant ID: ☐☐ ☐☐☐☐☐ ☐☐☐☐ **Date of Birth:** ____/___/____

Respiratory Viruses Causing Influenza-Like Illnesses in Healthy Australian Adults Aged 18 to 64 years: molecular and clinical characteristics:

Illness Visit Questionnaire

Dear Participant,

Thank you for agreeing to take part in our study to find out the range of viruses that cause respiratory infections. We are interested in knowing about the length and severity of illness experienced by people with different virus infections and how many people in your household were ill. **On the first day during recovery that you have only mild or no symptoms**, we would appreciate 5 minutes of your time to complete the following brief questionnaire. Please return the completed sheet in the reply paid envelope provided. If any questions are unclear and you need help to complete the form, please feel free to phone__________ on ___________

Date of illness onset ____/____/_____ Date of illness visit ____/_____/____

Please circle each symptom you experienced during this illness

Fever (≥ 37.8°C)	Feeling feverish	Chills	Muscle/body aches
Headache	Irritability	Decreased activity	Vomiting
Runny Nose	Blocked nose	Sore Throat	Cough
Wheezing	Shortness of breath	Moist cough, rattly chest	Pneumonia

Did you receive antivirals from study staff at your illness visit? Yes / No

Did you have to take time off from your usual activities (work, study, etc) during this illness? Yes / No

If yes, how many days? __________ days

Did you consult a health care provider during this illness? Yes / No

If yes, what kind (eg GP, district nurse, naturopath)? ______________________

If so, did your health care provider diagnose and/or treat you for any of the following conditions (please circle all that apply)?

Bronchitis / Pneumonia / Chest Infection / Ear Infection / Sinusitis

Were you admitted to a hospital ward for at least one night? Yes / No

If yes, Admission date _____/_____/_____ Discharge date _____/_____/_____

What day during recovery did you first experience only mild or no symptoms?
_____/_____/_____

Please complete the table below:

1. List details of the gender and age of all the people who live in your household, including you. If you need more rows, just write below the table.

2. Did anyone else in your household have a similar illness in the fortnight before you, or between the start of your illness and today? Please answer Yes or No for each person.

3. For each person who experienced a similar illness, please write the date on which their symptoms started in the far right column.

Person Number	Male or Female	Age (Years)	Was this person unwell? (Yes or No)	Date of illness onset (eg 01 June)
1 (You)				
2				
3				
4				
5				
6				
7				

Date you completed this questionnaire _____/_____/____

Thank you once again for your time and help with this study!

Yours sincerely

The Viral ILI Sub-study Investigators
